# SNP Analysis Infers that Recombination Is Involved in the Evolution of Amitraz Resistance in *Rhipicephalus microplus*

**DOI:** 10.1371/journal.pone.0131341

**Published:** 2015-07-09

**Authors:** Samantha Baron, Nicolaas A. van der Merwe, Maxime Madder, Christine Maritz-Olivier

**Affiliations:** 1 Department of Genetics, University of Pretoria, Pretoria, South Africa; 2 Department of Biomedical Sciences, Institute of Tropical Medicine, Antwerp, Belgium; 3 Department of Veterinary Tropical Diseases, University of Pretoria, Pretoria, South Africa; University of Minnesota, UNITED STATES

## Abstract

*Rhipicephalus microplus*, better known as the Asiatic cattle tick, is a largely invasive ectoparasite of great economic importance due to the negative effect it has on agricultural livestock on a global scale, particularly cattle. Tick-borne diseases (babesiosis and anaplasmosis) transmitted by *R*. *microplus* are alarming as they decrease the quality of livestock health and production. In sub-Saharan Africa, cattle represent a major source of meat and milk, but this region of the world is severely affected by the *Rhipicephalus microplus* tick. The principal method for tick control is the use of chemical acaricides, notably amitraz, which was implemented in the 1990’s after resistance to other acaricides surfaced. However, the efficiency of chemical control is hindered by an increase in the frequency of mutant resistance alleles to amitraz in tick populations. Presently, the only way to assess amitraz resistance is by means of larval packet tests, but this technique is time-consuming and not particularly cost effective. The main aims of this study were three-fold. First, we attempted to correlate two known SNPs in the octopamine/tyramine (OCT/Tyr) receptor with amitraz resistance in South African field samples of *R*. *microplus*. Second, we calculated gametic disequilibrium for these SNPs to determine whether they are randomly associated. Lastly, we conducted a study to assess the evolutionary effects of recombination within the OCT/Tyr receptor. Our results confirmed that the two SNPs are associated with amitraz resistance in the South African tick strain, and that they are in gametic disequilibrium. Additionally, recombination was detected in the OCT/Tyr receptor generating two recombinant haplotypes. These results are of concern to farmers in sub-Saharan Africa, and the emergence of amitraz resistance should be closely monitored in future. Therefore, we present a quick and affordable RFLP based diagnostic technique to assess amitraz resistance in field samples of *R*. *microplus*.

## Introduction

*Rhipicephalus microplus* ticks are hematophagous ectoparasites of veterinary importance, and are capable of parasitizing a variety of hosts, although cattle are their primary preference [1]. These ticks are adept in transmitting a variety of tick-borne pathogens to cattle, most notably *Babesia bovis*, which causes Asiatic babesiosis or redwater [1, 2]. The lack of efficient tick control strategies and management programs results in a severe economic burden, threatening the sustainability of the livestock industry in South Africa and globally. The use of chemical acaricides is still the most preferred method for tick control, but has become less effective due to the emergence of resistance. To date, resistance in ectoparasites has been reported for all the major classes of acaricides, including synthetic pyrethroids [3–8], organophosphates and carbamates [9–11], formamidines [11–14] and macrocyclic lactones [13, 15, 16]. Resistance to acaricides has been attributed to target site insensitivity as well as metabolic detoxification, depending on the mode of action of the acaricide.

*Rhipicephalus microplus* ticks have acquired the ability to evade the toxic effects of chemical acaricides by developing different resistance mechanisms. The cuticle surrounding the tick, which reduces acaricide access to the internal environment of the tick body, confers penetration resistance. However, further investigations into this type of resistance in *R*. *microplus* has not been reported since 1983 [17]. An additional resistance mechanism common in arthropods is target site insensitivity. This adaptive mechanism involves the alteration of the drug target site at the DNA level by alteration of the wild-type allele to a mutant form, which renders acaricide treatment ineffective. Lastly, metabolic resistance to acaricide treatment involves the increased ability to detoxify or sequester the acaricide. This involves the up-regulation of common detoxifying enzymes including cytochrome P450s, esterases and glutathione-S-transferases [11]. All of the aforementioned mechanisms have been shown to play a vital role in tick resistance to chemical acaricides.

Amitraz is a common formamidine acaricide, which is extensively used for tick control in South Africa. The target site for amitraz in *R*. *microplus* has yet to be defined, which ultimately delays any further development with regard to screening assays for diagnostics. It was proposed that monoamine oxidase, alpha-2-adrenceptors, and the octopamine receptor are good candidates for potential target sites, with the latter being the most probable in ticks [18]. It is thought that amitraz is a potential agonist of the octopaminergic system located in the tick synganglion. It has been suggested that in the presence of amitraz, the octopamine receptor is activated and this overstimulation at synapses has lethal effects on the tick [19]. The octopaminergic receptors have been classified into three distinct classes, namely α-adrenergic-like (αOCT), β-adrenergic-like (βOCT), and octopamine/tyramine (OCT/Tyr) or tyraminergic [20].

Resistance to amitraz is complex and suggested to be multigenic in nature, involving recessive inheritance of resistance alleles [21–23]. To date, no adequate resistance mechanisms against amitraz have been shown for *R*. *microplus*, but several such mechanisms have been proposed. Li, Davey [23] illustrated, by means of synergistic studies with enzyme inhibitors, that metabolic detoxification plays a role in amitraz resistance. However, these results were variable across different *R*. *microplus* strains, and the overall contributions of detoxifying enzymes were difficult to evaluate. The Mexican Pesqueria tick strain was confirmed to convey metabolic resistance to amitraz by up-regulation of glutathione-S-transferase [24]. It was further suggested that target site insensitivity could perhaps be the main mechanism of amitraz resistance. Unfortunately, studies to illustrate this mechanism have been unsuccessful [11, 22].

Baxter and Barker [25] sequenced the putative octopamine receptor from amitraz resistant and susceptible *R*. *microplus* Australian strains. Sequence analysis revealed no differentiation between the two phenotypes. It was later proposed that two single nucleotide polymorphisms (SNPs) in the octopamine receptor were linked to amitraz resistance [26]. However, these SNPs were inferred only by sequence alignment between a susceptible Australian strain, the susceptible American Gonzalez strain, and the resistant Santa Luiza strain [26]. Sequencing of the octopamine receptor gene from these strains revealed 37 SNPs, of which nine were non-synonymous substitutions. Seven of these were attributed to geographical differences between the strains, while the remaining two SNPs were potentially linked to amitraz resistance. These two SNPs occur at amino acid position 8 (threonine to proline) and 22 (leucine to serine) of the octopamine receptor protein [26]. Recent studies indicated that previous work was most likely conducted on a OCT/Tyr-like receptor rather than the native octopamine receptor [27]. This consequently led to the discovery of a SNP (*I61F*) in the β-adrenergic-like octopamine receptor of *R*. *microplus* [28]. This particular SNP was only localized to the central Queensland (Australia) region and absent in north and southeast regions, thus minimizing its probability of being the major causative reason for amitraz resistance in Australia. However, it should be noted that the Australian *R*. *microplus* tick strain might be a separate species re-classified as *R*. *australis* [29, 30]. Currently, the only way to effectively evaluate amitraz resistance is by means of larval packet tests (LPTs) [31]. However, these assays are limited in practicality as it takes approximately six weeks to obtain results.

The aim of this study was to evaluate published SNPs in the OCT/Tyr receptor [26] in South African field populations of *R*. *microplus*. Amitraz resistant and susceptible larvae from a controlled, closed off area in the Kruger National Park in South Africa were screened for these two SNPs. A correlation was then made between the presence of these SNPs and LPT results. Consequently, a country-wide survey to detect the presence of these SNPs was conducted. In order to better understand the complex nature of amitraz resistance in ticks, further analysis was done to test for the presence of gametic disequilibrium between these SNPs, and to investigate the contribution of recombination towards the emergence of amitraz resistance. Finally, the results from these analyses led to the development of an RFLP based diagnostic tool for assessing amitraz resistance in South African tick populations.

## Results

### Screen for SNPs in amitraz resistant and susceptible larvae

**Fig 1:**
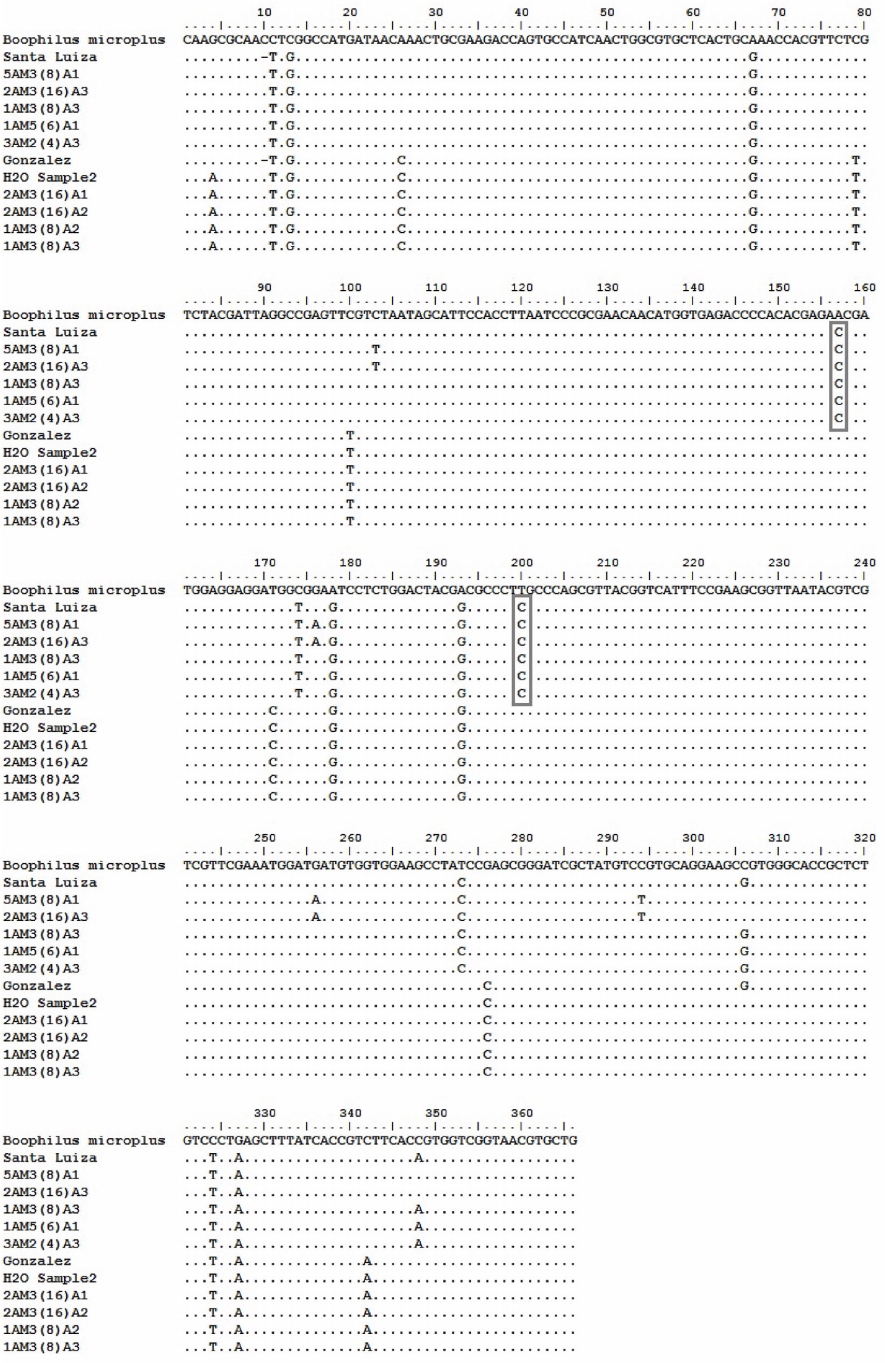
Sequence alignment of OCT/Tyr receptor gene fragment for amitraz resistant and susceptible *R*. *microplus* larvae. *Rhipicephalus (Boophilus) microplus* was the reference sequence used for the alignments (GenBank Accession: AJ010743.1) along with the Santa Luiza resistant strain (GenBank Accession: EF490688.1) and the Gonzalez susceptible strain (GenBank Accession: EF490687.1). The five samples aligned below the Santa Luiza strain are the resistant samples, and those below the Gonzalez strain are the susceptible ones. The grey blocks indicate the two resistance-associated SNPs. GenBank accession numbers for all samples are shown in S1 Table.

**Fig 2:**
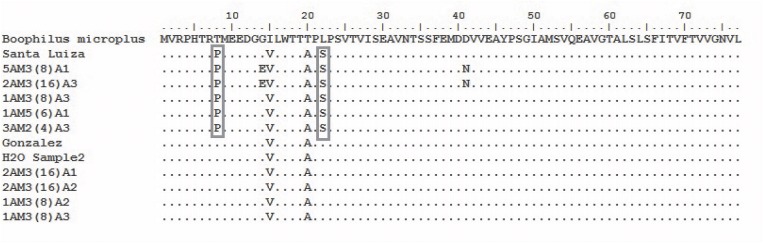
Amino acid sequence of a portion of the OCT/Tyr receptor gene. The grey blocks indicate the two resistance associated SNPs (T8P and L22S).

Amitraz resistant *R*. *microplus* larvae obtained from the Mnisi area in the Kruger National Park were screened for the presence of the two resistant SNPs published by Chen, He [26]. Twenty-four nucleotide substitutions were detected among resistant larvae, susceptible larvae, and the three reference strains from NCBI (Fig 1). Seven of the substitutions appeared to be associated with susceptible samples. The first four of these substitutions occurred in the non-coding region (nucleotide position 1–135) of the gene consisting of one transversion and three transition mutations. The three remaining substitutions within the open reading frame were synonymous, having no observable effect on the amino acid sequence. Four substitutions occurred in all resistant samples (Fig 1) and contained two non-synonymous sites at nucleotide positions 157 and 200 (Fig 2, T8P and L22S). These two substitutions corresponded with the two published SNPs. There were seven nucleotide substitutions differentiating *Rhipicephalus* (*Boophilus*) *microplus* G-protein coupled receptor (GenBank Accession AJ010743.1) from the other sequences, three of which occurred in the non-coding region and two non-synonymous changes in the coding region (I15V and T20A) (Fig 2). Lastly, there were several other SNPs that seemed to appear, with two non-synonymous substitutions occurring at nucleotide positions 176 and 256 (Fig 1). Samples containing these additional SNPs were amitraz resistant. GenBank accession numbers for all sequences obtained is shown in S1 Table.

The sequence data obtained for all larvae were compared to the results from LPT assays (Table 1). The comparison revealed a direct correlation between the phenotypic and suggested genotypic amitraz resistant status of sequenced larvae. The comparison was primarily made between larvae that survived the LPT assay (resistant) and those that did not (susceptible). Results showed that all larvae that did not survive the LPT assay displayed the AA/TT wild type genotype, or the heterozygous AC/TC genotype at the two SNP positions published by Chen, He [26]. Larvae that survived the amitraz exposure displayed the CC/CC mutant genotype.

**Table 1:** Correlation between phenotypic (LPT) and genotypic (sequencing) amitraz resistance status.

	**Larvae that survived LPT** ^a^		
**Sample**	**RF of strain** ^c^	**Genotype** ^d^	**Phenotype**
5AM2(4)A2	100	CC/CC	Amitraz Resistant
5AM2(4)A3	100	CC/CC	Amitraz Resistant
5AM3(8)A1	100	CC/CC	Amitraz Resistant
5AM3(8)A3	100	CC/CC	Amitraz Resistant
3AM2(4)A3	100	CC/CC	Amitraz Resistant
2AM3(16)A3	28	CC/CC	Amitraz Resistant
1AM3(8)A3	10	CC/CC	Amitraz Resistant
	**Larvae that did not survive LPTs** ^b^		
**Sample**	**RF of strain** ^c^	**Genotype** ^d^	**Phenotype**
2AM3(16)A1	28	AA/TT	Amitraz susceptible
2AM3(16)A2	28	AA/TT	Amitraz susceptible
1AM3(8)A2	10	AA/TT	Amitraz susceptible
1AM3(8)A3	10	AA/TT	Amitraz susceptible
1AM3(8)A1	10	AC/TC	Amitraz susceptible
1AM3(16)A1	10	AC/TC	Amitraz susceptible

Discriminating dose of amitraz used was 250 ppm.

^a^ Larvae that were alive after the larval packet test assay was completed, meaning they were resistant to the amitraz concentrations applied.

^b^ Larvae that were susceptible to the concentrations of amitraz used and were dead after the assay was completed.

^c^ RF is the resistance factor, a RF of 100 implies that the strain is amitraz resistant. RF of 10 and 28 means the strain is susceptible to amitraz.

^d^ Genotype was inferred based on whether or not the sequenced larvae contained the two SNPs published by Chen, He (26). CC/CC – all four alleles are resistant (homozygous resistant), AA/TT – all four alleles are the wild type alleles (homozygous susceptible), AC/TC – at both SNP positions both the wild type and mutant alleles are present (heterozygous).

### Country-wide screen for SNPs in adult ticks

Adult ticks from 108 different farms showed a 30% incidence of *R*. *microplus*, equating to a total of 33 farms that were used in this study. These 33 farms resided in five different provinces across South Africa. The majority of the ticks collected were from the Kwa-Zulu Natal Province (S1 Fig). Therefore, a total of 218 alleles per published SNP (109 adult *R*. *microplus* ticks, approximately three per farm) were screened.

Several additional SNPs were found within the field samples across South Africa, mainly within the non-coding region of the OCT/Tyr receptor gene (data not shown). The frequencies of susceptible (AA/TT) and resistant (CC/CC) genotypes were calculated for the resistance-linked SNPs occurring in the OCT/Tyr receptor gene for all adult ticks (Table 2). The majority of the population exhibited the heterozygous (AC/TC) genotype at these nucleotide positions, with half of the population displaying the resistance C allele for amitraz. The individual genotype for each *R*. *microplus* tick sample is shown in S2 Table.

**Table 2:** Genotype and allele frequencies for *R*. *microplus* tick samples across South Africa.

**Genotype Frequencies**		
**Genotype**	**Occurrence**	**Frequency**
AA/TT	26	0.2385
AC/TC	62	0.5688
CC/CC	21	0.1927
**TOTAL**	**109**	**1**
**Allele Frequencies (SNP1)** ^a^		
**Allele**	**Occurrence**	**Frequency**
A allele	114	0.5229
C allele	104	0.477
**TOTAL**	**218**	**1**
**Allele Frequencies (SNP2)** ^b^		
**Allele**	**Occurrence**	**Frequency**
T allele	114	0.5229
C allele	104	0.477
**TOTAL**	**218**	**1**

^a^ Refers to the SNP that occurs at nucleotide position 157 (A-C)

^b^ Refers to the SNP that occurs at nucleotide position 200 (T-C).

### Characterization of heterozygous mutants

**Fig 3:**
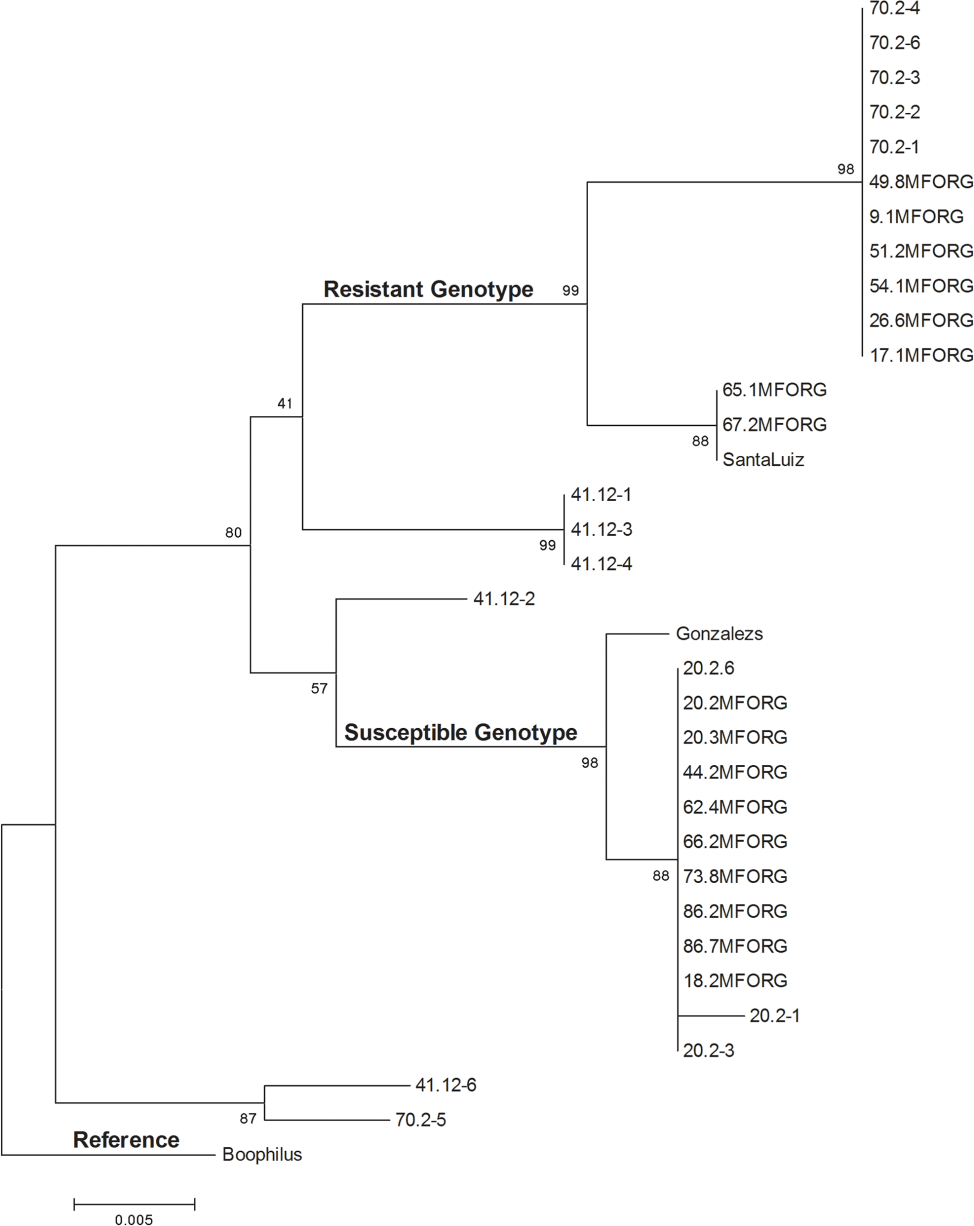
Lineages based on OCT/Tyr receptor sequences from *R*. *microplus* ticks in South Africa. The evolutionary history was inferred using the Neighbor-Joining method [56]. The optimal tree with the sum of branch length = 0.10881934 is shown. The percentage of replicate trees in which the associated taxa clustered together in the bootstrap test (500 replicates) are shown next to the branches [59]. The tree is drawn to scale, with branch lengths in the same units as those of the evolutionary distances used to infer the phylogenetic tree. The evolutionary distances were computed using the Maximum Composite Likelihood method [60] and are in the units of the number of base substitutions per site. The analysis involved 34 nucleotide sequences. All positions containing gaps and missing data were eliminated. There were a total of 364 positions in the final dataset. Evolutionary analyses were conducted in MEGA5 [55]. GenBank accession numbers for each sample is displayed in S1 Table.

Heterozygous individuals grouped into three classes based on their preliminary sequence data. One individual fully representing each class was chosen for further characterization. Cloning and sequencing from phenotypically heterozygous individuals revealed allelic combinations across all loci. Comparisons between sequenced clones, homozygous individuals and the three reference strains from NCBI demonstrated ten distinct lineages, of which eight occur in South Africa. Fig 3 represents the ten different lineages based on sequence variants. Two distinct lineages represented the resistant ticks with their segregation supported by a bootstrap value of 99. A poor bootstrap value [41] separated the two resistant lineages from characterized heterozygous individuals containing the SNP at nucleotide position 157 (*i*.*e*. clones 41.12). A bootstrap value of 80 separated the resistant individuals and the 41.12 (-1, -3 and -4) clones from the susceptible individuals, suggesting that the three clones are closely related to the resistant strains. Heterozygous individuals displayed sequence characteristics of both susceptible and resistant genotypes, making the interpretation of these genotypes difficult. In field samples it was especially challenging due to the presence of additional SNPs throughout the gene fragment.

### Gametic disequilibrium between SNPs

**Fig 4:**
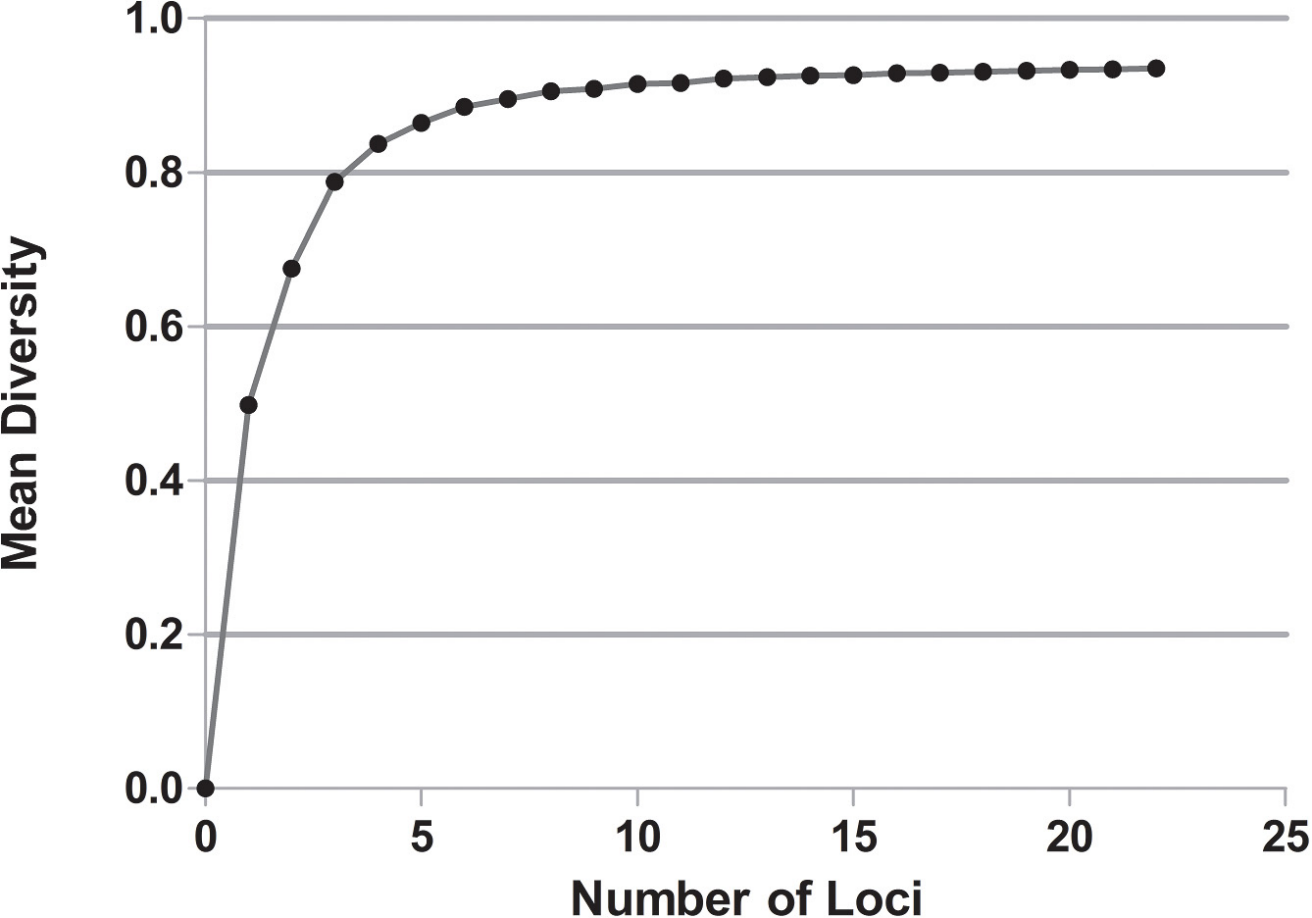
Distribution of the mean diversity versus the number of loci for the OCT/Tyr receptor. A comparison of 22 different loci within the OCT/Tyr receptor is shown. The plateau of mean diversity implies that there are sufficient samples for further analysis, and the addition of more samples or loci will not change the diversity observed.

In this study, all SNPs that were present in the OCT/Tyr receptor were analyzed to determine whether they were in gametic disequilibrium. First, diversity was modeled against the number of loci (variable positions in the sequences) (Fig 4). This is essential to determine if there are sufficient samples for the proposed analysis. The graphical plot of mean diversity versus the number of loci should reach a plateau, signifying that there are sufficient samples for further analysis (which is evident in Fig 4). Thus, additional samples or loci would not have changed the diversity that was observed in the sample set.

**Fig 5:**
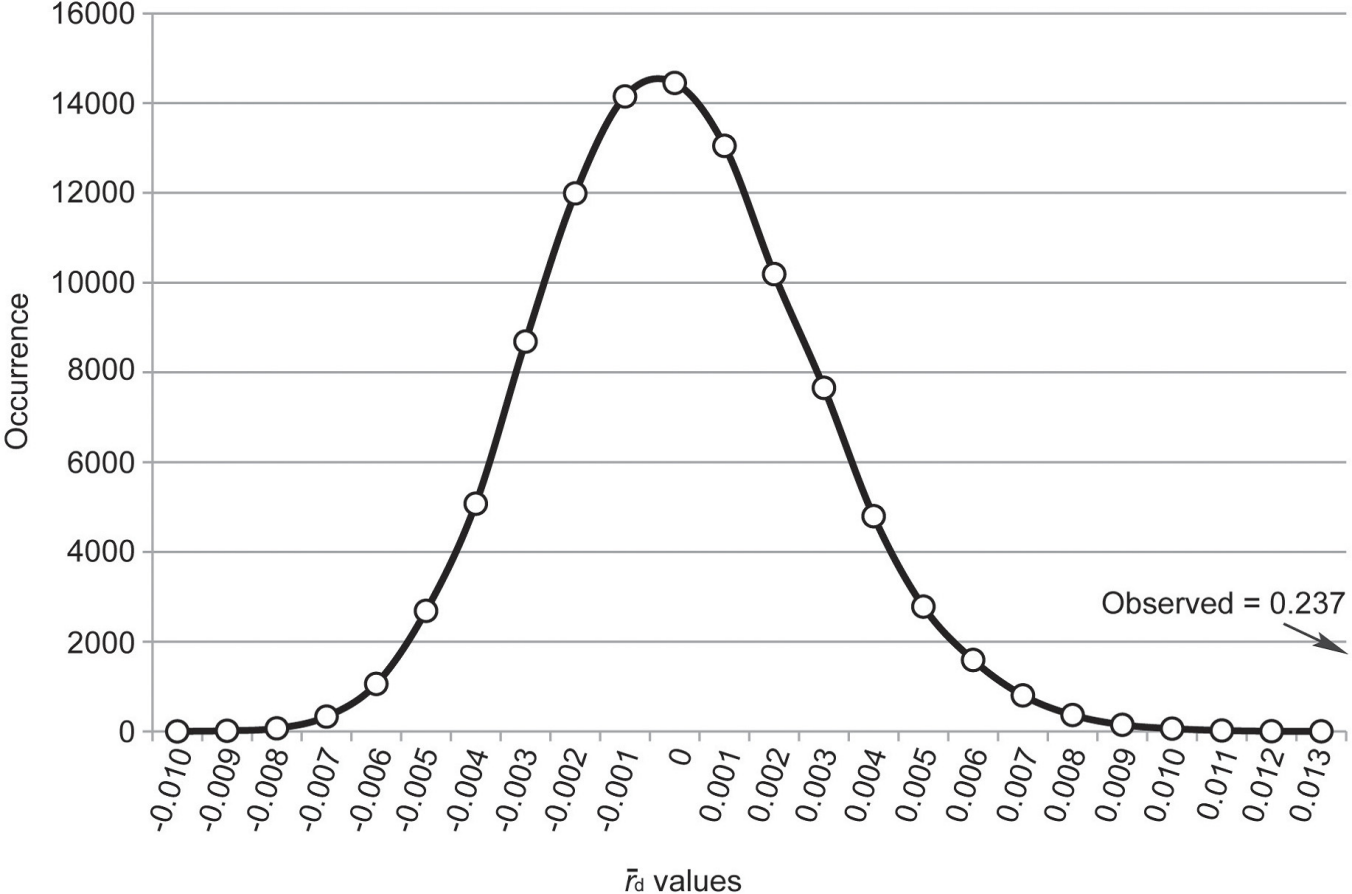
The ${\bar{r}}_{d}$ density distribution for the OCT/Tyr receptor. The ${\bar{r}}_{d}$ values are placed on the x-axis while the relative occurrence of each of these values is displayed on the y-axis. The observed value falls outside of the distribution range generated by the randomized data set indicating that there is gametic disequilibrium.

An analysis of gametic disequilibrium resulted in a graphical plot with a bell shaped curve, with the observed ${\bar{r}}_{d}$ value falling outside of the general distribution (Fig 5). This implies that the observed ${\bar{r}}_{d}$ value was significantly higher (*P* < 0.00001; *H*_*0*_ = random association or gametic equilibrium) than what was generated by the randomized data set, thus emphasizing a deviation from random association. Therefore, the observed data displayed a significant level of gametic disequilibrium (non-random association), while gametic equilibrium (random association) was absent.

Initial comparisons were done for the two SNP loci that were shown to be involved in amitraz resistance (loci 11 and 17). Locus 11 corresponds to nucleotide position 157, and locus 17 with that of 200. A pair-wise comparison of these two loci resulted in a ${\bar{r}}_{d}$ value of 0.804 (*P* < 0.00001), indicating that these two sites are in gametic disequilibrium. Resistant samples showed that both substitutions were present (CC/CC genotype), indicating that the two loci are strongly associated.

Pair-wise comparisons were performed for all variable loci within the gene to identify the loci that were significantly associated with the two resistant loci (Table 3). Locus 12 corresponds to nucleotide position 171 in the OCT/Tyr receptor alignment, locus 13 to that of 174, and locus 19 to that of 273 (Fig 1). The aggregate value of ${\bar{r}}_{d}$ was calculated to determine which of the three loci were most strongly associated with the two resistance loci (11 and 17). The two resistant loci were equally associated with loci 12 and 13. When comparing sequence data, it was evident that a nucleotide substitution at locus 12 only took place when the tick was suspected to be susceptible to amitraz. A substitution at locus 13, however, occurred every time the tick was suspected to be resistant. As a result, this particular association was considered as a potential molecular marker for amitraz resistance in the OCT/Tyr gene.

**Table 3:** Loci significantly associated with resistant loci 11 and 17.

SNP 1	SNP 2	${\bar{r}}_{d}$ value^a^	Aggregate^b^	P-value
Locus 11	Locus 12	0.834	0.877	< 0.00001
Locus 17	Locus 12	0.921	0.877	< 0.00001
Locus 11	Locus 13	0.872	0.878	< 0.00001
Locus 17	Locus 13	0.884	0.878	< 0.00001
Locus 17	Locus 19	0.803	0.803	< 0.00001

^a^ ${\bar{r}}_{d}$ value represents the gametic disequilibrium that is observed between the two SNPs being compared to one another.

^b^ Aggregate value is the mean value of ${\bar{r}}_{d}$ values that were obtained for loci 11 and 17 associated with the other loci to determine which association displayed the most gametic disequilibrium.

### Recombination in the OCT/Tyr receptor gene

**Fig 6:**
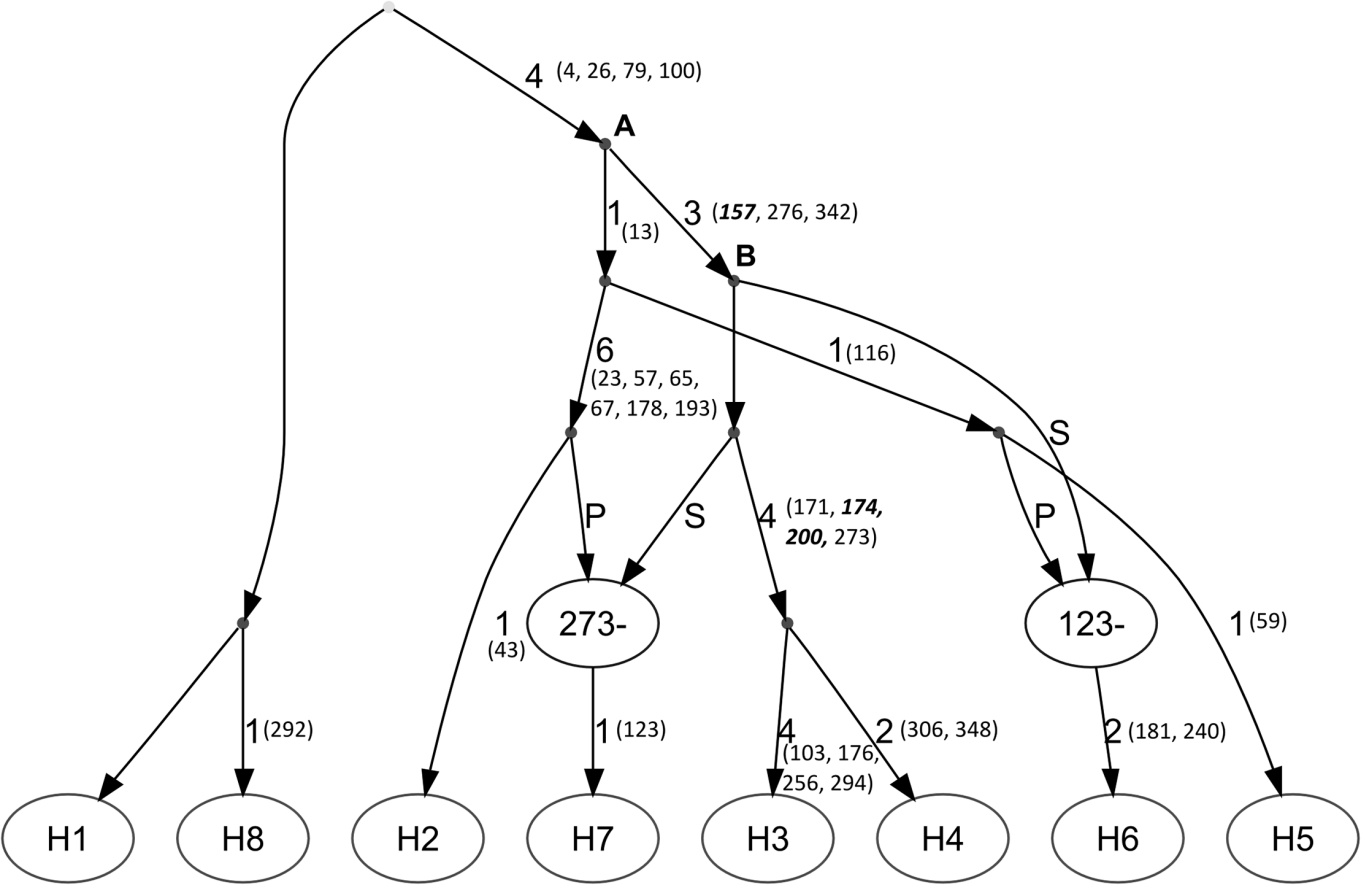
Ancestral recombination graph for homozygous amitraz resistant and susceptible *R*. *microplus* ticks in South Africa as well as characterized heterozygotes. H1 to H8 represent the infinite-sites-compatible haplotype sequences that were observed. Two recombination events were detected generating two recombinant haplotypes (H6 and H7). The H6 haplotype represented an intermediate (or heterozygous) state containing the SNP at nucleotide position 157 but not at 200. The two resistance-linked SNPs are shown in bold along with site 174 which is the potential biomarker for amitraz resistance. The numbers in brackets represent the nucleotide positions where mutations occurred within that haplotype. GenBank accession numbers and haplotype designations are shown in S1 Table.

An ancestral recombination graph (ARG) was constructed by incorporating sequence information from the OCT/Tyr receptor gene of resistant (CC/CC) and susceptible (AA/TT) South African *R*. *microplus* ticks as well as the cloned and characterized heterozygotes (Fig 6). The ARG illustrated the presence of eight South African haplotypes, substantiating previous observations (Fig 3), with two recombination events. The H3 and H4 haplotypes represent the two resistant strains in South Africa, and their accumulation of resistance-associated SNPs are indicated by bold italic numbers in Fig 6. The H6 haplotype represents the characterized heterozygous individuals which contain the SNP at nucleotide position 157 but not at 200. The remaining haplotypes were amitraz susceptible. The resistant H3 and susceptible H2 haplotypes coalesce at position A, indicating the point of divergence. Coalescent point B indicates the divergence of H3 from the heterozygous H6 haplotype. The SNP at nucleotide position 157 is retained in the H6 haplotype through a recombination event which utilizes the suffix of H3 after nucleotide position 123. An additional recombination event occurs to generate the susceptible H7 haplotype. No geographical significance was found with the haplotype-sample groupings or the areas in South Africa where the samples originated.

### RFLP based diagnostic tool for amitraz resistance

Locus 13, corresponding to nucleotide position 174, was significantly associated with the two resistant loci in the OCT/Tyr receptor gene (Table 3). This nucleotide substitution (C174T) is synonymous and occurred whenever the tick displayed both phenotypic and genotypic resistance. It also occurred in samples having the heterozygous (AC/TC) genotype. Consequently, this tight association between loci was investigated as a potential molecular marker for amitraz resistance.

**Fig 7:**
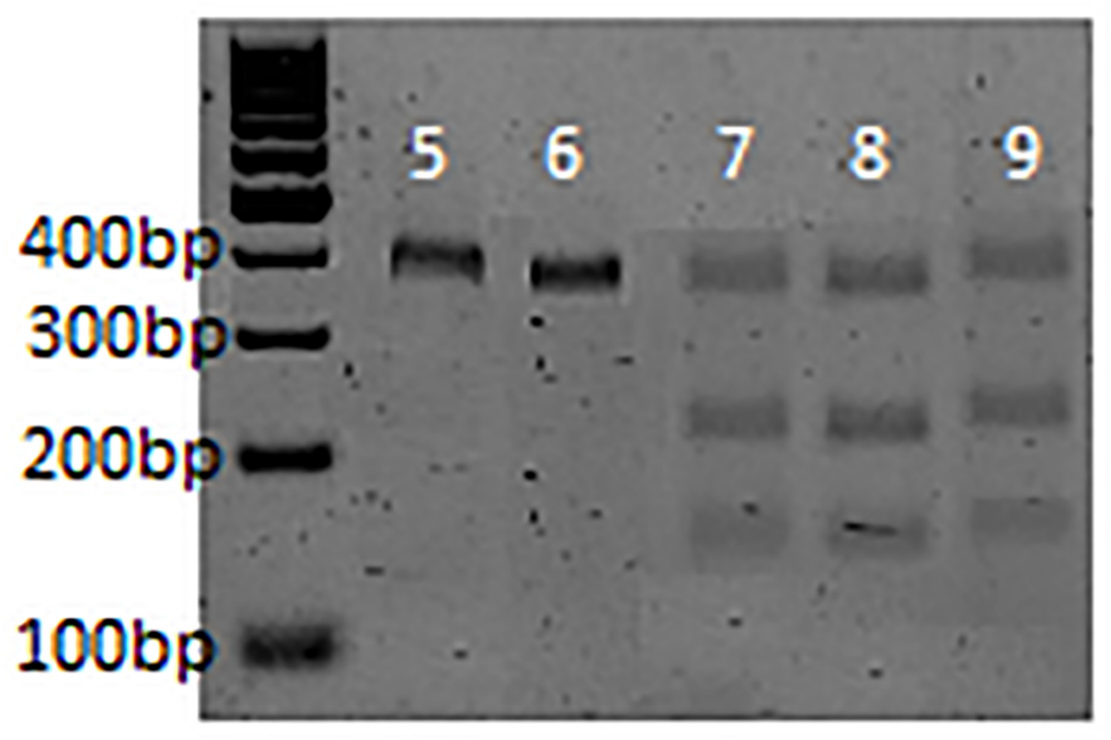
Gel electrophoresis of the OCT/Tyr receptor from *R*. *decoloratus* larvae digested with *Eci*I restriction enzyme. The lane numbers represent samples from the original set of 14 anonymous samples. Lane 1-sample 5, lane 2-sample 6, lane 3-sample 7, lane 4-sample 8 and lane 5– sample 9 (S3 Table). A single band at 400 bp indicates a homozygous resistant genotype and phenotype. The presence of additional bands at 223 and 186 bp indicates that the sample is heterozygous and susceptible to amitraz treatment.

*R*. *decoloratus* ticks with known amitraz resistance and susceptibility were used to test the effectiveness of the restriction enzyme *Eci*I as a possible diagnostic tool. The *Eci*I enzyme recognizes 5’ - GGCGGA(N11/9) – 3’ making it an efficient enzyme to detect a nucleotide substitution at position 174. Fourteen anonymous samples, each containing multiple larvae was provided by Ms Ellie van Dalen, University of the Free State. Of these, five samples were used for RFLP analyses (Fig 7). Due to the strong association between loci, a genotype of TT at nucleotide position 174 would correspond to the CC/CC resistant genotype.

Sample 5 (Lane 1, Fig 7) showed a prominent band at 400 bp, indicating a TT genotype and resistant phenotype. This result was corroborated by an independently performed larval packet test (S3 Table). This particular sample exhibited 13.2% control at a field concentration of 250 ppm amitraz. Sample 6 (Lane 2, Fig 7) showed resistance potential in the same fashion as Sample 5, and this was corroborated by a 30% level of control during independent larval packet tests (S3 Table). Samples 7, 8 and 9 (Lanes 3, 4 and 5 respectively, Fig 7) produced identical digest products. In addition to a 400 bp digest product, a 223 bp and 186 bp product was also detected. This result signifies that these samples were heterozygous (CT genotype at position 174) and were potentially more susceptible to amitraz treatment. Indeed, independent larval packet tests revealed that these samples were 100% susceptible to field concentrations of amitraz (S3 Table).

## Discussion

In this study, we have investigated the evolution of amitraz resistance in South African *Rhipicephalus microplus* ticks. Two previously known SNP loci in the OCT/Tyr receptor [26] were significantly associated with each other, and with amitraz resistance. Lastly, we exploited the high level of gametic disequilibrium between these two SNPs and an additional SNP, which changes a restriction enzyme binding site, in order to devise an affordable an easy restriction enzyme based assessment tool for amitraz resistance in field samples of ticks.

The octopaminergic receptors have been classified into three classes. The α-adrenergic-like octopamine receptors display an increased affinity for octopamine rather than tyramine, which leads to an increase in intracellular Ca^2+^ concentrations together with a small increase in intracellular cAMP levels [32, 33]. The β-adrenergic-like octopamine receptors on the other hand are specifically activated in response to octopamine, directly resulting in increased intracellular cAMP levels [33, 34]. Lastly, the octopaminergic/tyraminergic (OCT/Tyr) receptors have shown similarity in terms of structure and pharmacology with the vertebrate α2-adrenergic receptors [20]. Agonistic preferences can result in these receptors being stimulated by either octopamine or tyramine. In response to octopamine, there will be an increase in intracellular Ca^2+^ concentrations. Conversely, a response to tyramine will result in a decrease in intracellular cAMP levels [33]. Robb, Cheek [35] also demonstrated this principle in *Drosophila*, where one receptor can display different pharmacological profiles with regard to second messenger systems implemented.

Previous studies done in honeybees [36] and mammals [37] suggested that the proposed target site for amitraz is the α-adrenergic-like receptors and the α2-adrenergic receptors respectively. This, along with the SNPs discovered by Chen, He [26] strongly suggest the involvement of OCT/Tyr like receptors in amitraz resistance. The mutation reported by Corley, Jonsson [28] in the β-adrenergic-like octopamine receptor seems promising, but its restricted localization to only the central part of Queensland in Australia further substantiated our notion to investigate the OCT/Tyr receptor instead. Additionally, it has been reported that *R*. *microplus* in Australia may be a different species and has therefore been re-classified as *R*. *australis* [29, 30].

A country-wide screening for the two resistance-associated SNPs in the OCT/Tyr receptor revealed an elevated frequency (0.5688) of the heterozygous AC/TC genotype. We hypothesize that positive balancing selection is acting on the population in order to maintain the prevalence of heterozygosity [38]. Selection pressure is imposed on the tick population by the application of amitraz, and this drives resistance-conferring alleles to homozygosity [12, 39]. On the other hand, the potential advantages of heterozygosity without amitraz selection pressure can cause subsections of the population, which escape amitraz treatment, to remain heterozygous. This observation could be further explained by a slow rate of fixation of amitraz resistance in comparison to other acaricides [21]. Studies also suggested that there is a lack of fitness in amitraz-resistant strains, thus providing insight into the possible selective disadvantage that may be associated with homozygous resistant ticks [40]. This particular fitness cost would largely contribute to the excess heterozygotes found within the current study. Such interactions between selection pressure and fitness cost will determine the observed resistance-associated allele frequency, and may occur through direct or pleiotropic effects [41].

The two putative resistance-associated SNP loci were in gametic disequilibrium and they were positively associated with the resistant phenotype. Thus, if both SNP loci display specific nucleotide substitutions, it can be inferred that the tick is resistant to amitraz. Although significant gametic disequilibrium exists for these SNPs, there is a small but important deviation from complete disequilibrium. A decay in gametic disequilibrium among loci can be generated from a variety of factors, namely mutation, population admixture, gene flow, genetic drift, diversifying natural selection as well as genetic heterogeneity [42, 43]. Coalescent interaction of these forces within the tick population are likely responsible for the incomplete disequilibrium observed here.

A third SNP position in the OCT/Tyr receptor gene was significantly associated with the two previously mentioned SNPs. This suggests that amitraz resistance is potentially associated with complete alleles of the gene, instead of with individual SNP positions. Therefore, associations among SNP positions could potentially be attributed to intact inheritance of alleles. This is further demonstrated by additional variable positions that seemed to distinguish resistant from susceptible phenotypes. Upon constant selection pressure, these variable positions have probably been driven to fixation in the population. Therefore, it seems as though exposure to amitraz consequently affects a wider range of loci, further substantiating the probability of intact inheritance of alleles. A classic evolutionary process that could be responsible for such an observation is epistatic interaction, where this combination of SNP alleles within the gene could compensate for the fitness cost mentioned previously [44].

We further investigated whether intragenic recombination could be inferred within the OCT/Tyr receptor gene. A minimum of two recombination events occurred in order to generate the haplotypes observed in Fig 6. Considering the relatively high polymorphic nature of the OCT/Tyr gene, detecting recombination was expected [45]. Recombination in the OCT/Tyr receptor gene would allow new combinations of the prefix and the suffix of the gene, which could allow for new SNP combinations, as well as the emergence of double homozygotes in the population. Our results also showed that such double homozygotes displayed resistance to amitraz (H3 and H4). It appears as though the loci at nucleotide position 157 is involved in recombination to generate the heterozygous haplotype. It could be hypothesized that the homozygous states represent ‘genetic load’ in the population and thus heterozygotes are favoured. Therefore, it seems that under constant amitraz selection pressure, recombination in the OCT/Tyr receptor gene plays an important role in the emergence of resistance to this acaricide. Recombination may act to generate or decay allelic combinations [43], and if natural selection acted on these combinations of alleles, they may be retained in the population [42]. This explains the complete allele inheritance observed within this population. The relative strength of these two processes will determine the strength of the disequilibrium among loci [43]. It should be noted that discerning an ancestral state with recombination is rather difficult [46, 47], therefore it is not assumed that the H1 susceptible haplotype represents the common ancestor in this study.

Gametic disequilibrium will be transitory in the absence of amitraz selection pressure acting to maintain it, thus facilitating recombination which will break up nonrandom allelic associations [43, 48]. This could explain the additional variable sites that were detected in heterozygous genotypes which displayed minimal associations with other loci (data not shown). Thus, heterozygous sub-sections of the population could escape amitraz pressure, impeding disequilibrium and assisting recombination which gives rise to these new combinations of alleles.

Exploitation of the existence of associated SNPs resulted in accurate detection of amitraz resistance in anonymous samples. Molecular detection using a RFLP technique is more economical, easier and faster than traditional larval packet tests. Heterozygous (AC/TC) genotypes were characterized as susceptible, substantiating previous claims that amitraz resistance is recessively inherited [22]. The majority of the population displayed this heterozygous genotype. Extrapolation of the results obtained from the RFLP assessments suggested that this population is still susceptible to amitraz, with the exception of CC/CC resistant genotypes. Results of the recombination studies showed that the mutations at nucleotide position 174 and 200 arose before segregation of the H3 and H4 haplotypes. This indicates that resistance can be detected in different amitraz resistant strains with the same efficiency. This is apparent for the South African *R*. *microplus* tick population, but may not be the case in other global strains where amitraz resistance mechanisms may arise independently from what was observed in the current study. This protocol will play an important future role in monitoring the emergence of amitraz resistance in South African *R*. *microplus* and *R*. *decoloratus* ticks. Such information could aid in guiding the use of effective control chemicals, as well as predicting when and where amitraz resistance might appear. It is worthy to note that this procedure was conceded as a proof of concept approach, and additional verification will be done on a much larger sample size.

The novelty of this work includes the first confirmation of the two SNPs published by Chen, He [26] in amitraz resistant larvae, as well as field populations of ticks. It is also the first report of any association studies being conducted for the OCT/Tyr receptor in ticks. Recombination between non-synonymous SNP loci appears to be important in the generation of resistant phenotypes in the South African *R*. *microplus* population, but this hypothesis needs to be further investigated. Furthermore, a patent is currently being finalized for use of the RFLP based diagnostic tool to assess amitraz resistant field populations. Lastly, this study is the first indication that heterozygous field populations are susceptible to amitraz treatment, allowing for alternative and improved strategies to be implemented on farms before full blown resistance is acquired. This research paves the way forward for rapid diagnostics, and improvement of local vector control strategies to lessen the impact of anaplasmosis and babesiosis on human and animal health.

## Materials and Methods

### Tick collections, identification and DNA extraction

Adult ticks were collected by Zoetis (Pty) Ltd. from 108 different farms across South Africa, of which 33 farms contained *R*. *microplus* ticks (worked from an available field collection that was already established at the University of Pretoria, provided by Zoetis. Each farmer provided Zoetis with their consent for collection of ticks). Classification of collected ticks into their respective genera was done according to previous authors [1, 49]. Further differentiation between *Rhipicephalus (Boophilus)* species was done using microscopy techniques. To distinguish between *R*. *microplus* and *R*. *decoloratus* females, the hypostome dentition was examined, along with the adanal spurs for the male ticks [1, 49]. Molecular identification of ticks was also performed based on restriction fragment length polymorphism (RFLP) analyses [50].

Control samples of amitraz resistant larvae were obtained from the Mnisi area in Kruger National Park from Dr Rosalind Malan. Twelve different strains were provided, of which three were classified as resistant based on their resistance factors, which were determined by conventional larval packet tests [31].

A modified salt based extraction method published by Aljanabi and Martinez [51] was used for genomic DNA isolation from whole adult ticks (predominantly female ticks). Whole ticks were homogenized in 200 μl lysis buffer (0.5 M EDTA, 0.5% (w/v) Sodium lauroyl sarcosinate). An additional 400 μl of DNA extraction solution (0.4 M NaCl, 60 mM Tris-HCl, 12 mM EDTA, 0.25% SDS, pH 8.0) was added to the samples along with 2 μl of proteinase K (15 mg/ml), mixed well and incubated overnight at 55°C. Samples were incubated for 20 min at 65°C to inactivate the proteinase K, after which 1 μl of RNase A (10 mg/ml) was added. Samples were briefly vortexed and further incubated at 37°C for 15 min. Protein precipitation was performed by adding 360 μl of 5 M NaCl, vortexing for 10 sec, and incubation on ice for 5 min, followed by centrifugation at 25 500*xg* for 20 min at room temperature. An equal volume of isopropanol was added to the supernatant, briefly vortexed, followed by an incubation of 1 hour at -20°C. Samples were then centrifuged for 20 min at 10 000*xg* and the supernatants discarded. DNA pellets were washed three consecutive times with 500 μl of 70% ethanol, centrifuged for 5 min at 10 000*xg*, and the supernatant discarded. The final DNA pellets were air dried and then re-suspended in 50 μl of 1 x TE buffer (1 mM Tris-HCl, 0.1 mM EDTA, pH 7.0).

Genomic DNA was extracted from individual larvae using a modification of the protocol by Hernandez, Guerrero [52]. Briefly, individual larvae were crushed in a 1.5 ml microcentrifuge tube containing 25 μl TE buffer (10 mM Tris, 1 mM EDTA, pH 7.6). The suspension was then boiled for 5 min and centrifuged at 4000*xg* for 30 sec at room temperature. The supernatant was directly used for PCR.

### PCR amplification and sequencing of the OCT/Tyr receptor gene

Published primers by Chen, He [26] were used for PCR amplification of a 417 bp fragment of the OCT/Tyr receptor gene at an annealing temperature of 55°C. This method worked sufficiently for a few samples, but due to tick strain differences, a new forward primer was designed. The new forward primer, OAR-F172 (5’-AGC ATT CTG CGG TTT TCT AC-3’), was designed based on sequence data from the few samples that amplified successfully. This forward primer was used for the majority of the tick samples with the same reaction conditions.

All PCR products were sequenced by Macrogen Inc. (Netherlands) in a 96-well plate according to the standard dye terminator sequencing strategy. The sequences were analyzed using BioEdit sequence alignment editor version 7.2.0 [53], and multiple sequence alignments were performed using the online MAFFT program (http://mafft.cbrc.jp/alignment/software/) [54]. Sequences were aligned with published NCBI sequences to determine if any SNPs were present. The inferred amino acid sequences were used to identify synonymous and non-synonymous mutations.

### Cloning and sequence analysis of heterozygotes

PCR amplified OCT/Tyr receptor fragments (from heterozygous individuals) were cleaned using the QIAquick PCR Purification Kit (QIAGEN, USA), and ligated into the pGEM-T Easy Vector System (Promega, USA) according to manufacturer’s instructions. Ligation reactions were transformed into JM109 *E*. *coli* competent cells using electroporation. Six to eight individual clones per heterozygous individual was picked and grown in LB-Amp overnight at 37°C. The overnight cultures were processed using the Zyppy Plasmid Miniprep Kit (Zymo Research, USA) and cloned inserts were sequenced using the pUC/M13 forward and reverse sequencing primers (Inqaba biotec, South Africa). Sequences were analyzed using BioEdit and aligned with MAFFT against previously sequenced homozygous individuals. Evolutionary analysis of these sequences was performed in MEGA5 using the Neighbor-Joining method [55, 56].

### Gametic disequilibrium and ancestral recombination

Gametic disequilibrium studies were performed to determine intragenic association (linkage) between SNP alleles within the OCT/Tyr receptor gene. This analysis was carried out using the Multilocus 1.3b1 program [57]. For these analyses, 100 000 data randomizations were performed to compare the observed data with randomized data that mimic gametic equilibrium. If the observed dataset displayed increased gametic disequilibrium compared to the randomized datasets, it was assumed that there is association between the loci. This was further supported by *P*-values. Functions carried out using Multilocus included analysis of genotypic diversity versus the number of loci and linkage disequilibrium.

To determine the evolutionary histories within resistance genes, ancestral recombination graphs were constructed using SNAP Workbench [58]. Sequence alignments were converted into haplotypes by excluding indels and infinite site violations. The branch and bound Beagle algorithm in SNAP Workbench was implemented to infer the minimal number of recombination events within the gene that could explain the data [46].

### RFLP based diagnostic assay

The rapid diagnostic test for amitraz resistance was evaluated in the form of a RFLP analysis. Susceptible and resistant *R*. *decoloratus* larvae (confirmed through larval packet tests) were obtained from the University of the Free State, South Africa (Ms. Ellie van Dalen), for the purpose of a double blind study. Larvae were labeled from one through 14, with the status of their resistance unknown until after the experiment. The OCT/Tyr receptor gene was amplified from bulked larval DNA, and amplicons were subsequently treated with 4 U of *Eci*I restriction enzyme. Depending on the particular pattern that was observed after digestion, samples were classified as susceptible or resistant to amitraz treatment. These tests were compared to independently assessed resistance statuses in order to confirm the accuracy of the diagnostic test.

## Supporting Information

S1 Fig Subpopulation structure of ticks across South Africa. Ticks from each farm were placed into subpopulations (1–15) depending on the region from which they were collected. Grid blocks were constructed 300 x 300 km over the country for accurate overall segregation of populations. The farms from which tick samples were analyzed are indicated in the table, along with their grid block number and province. Farm numbers correspond with sample number, e.g. sample 44.1MF is sample 1 of female *R*. *microplus* from farm 44.
(DOCX)

S1 Table GenBank accession numbers for all *R*. *microplus* OCT/Tyr receptor sequences. (DOCX)

S2 Table Genotypes of field samples of *R*. *microplus* ticks at the two published SNP positions. (DOCX)

S3 Table *Rhipicephalus microplus* larval packet test results. (DOCX)
