# Supplementary material for: SNP Analysis Infers that Recombination Is Involved in the Evolution of Amitraz Resistance in Rhipicephalus microplus
Source: PLoS One. 2015 Jul 9;10(7):e0131341. doi: 10.1371/journal.pone.0131341 (PMC4497657; doi:10.1371/journal.pone.0131341)
Supplement: S1 Fig — Ticks from each farm were placed into subpopulations (1–15) depending on the region from which they were collected. Grid blocks were constructed 300 x 300 km over the country for accurate overall segregation of populations. The farms from which tick samples were analyzed are indicated in the table, along with their grid block number and province. Farm numbers correspond with sample number, e.g. sample 44.1MF is sample 1 of female R. microplus from farm 44. (DOCX) [file pone.0131341.s001.docx]

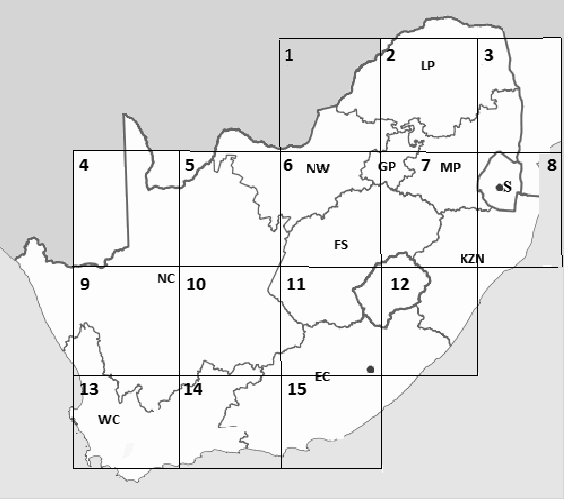


| **Farms** | **Grid block number** | **Province** |
| --- | --- | --- |
| 44, 46, 71 | 2 | Mpumalanga (MP) |
| 17 | 3 | Swaziland (S) |
| 26, 47, 86, 45 | 7 | Mpumalanga (MP) |
| 20, 54, 73 | 7 | Kwa-Zulu Natal (KZN) |
| 21, 50, 51 | 8 | Kwa-Zulu Natal (KZN) |
| 66, 67, 69 | 8 | Swaziland (S) |
| 9, 77 | 11 | Eastern Cape (EC) |
| 37, 40, 41, 49, 58, 62, 65, 70 | 12 | Kwa-Zulu Natal (KZN) |
| 7, 93, 95 | 12 | Eastern Cape (EC) |
| 18, 42 | 13 | Western Cape (WC) |
| 79 | 15 | Eastern Cape (EC) |

**S1 Fig. Subpopulation structure of ticks across South Africa.** Ticks from each farm were placed into subpopulations (1-15) depending on the region from which they were collected. Grid blocks were constructed 300 x 300 km over the country for accurate overall segregation of populations. The farms from which tick samples were analyzed are indicated in the table, along with their grid block number and province. Farm numbers correspond with sample number, e.g. sample 44.1MF is sample 1 of female *R. microplus* from farm 44.
