# Supplementary material for: SNP Analysis Infers that Recombination Is Involved in the Evolution of Amitraz Resistance in Rhipicephalus microplus
Source: PLoS One. 2015 Jul 9;10(7):e0131341. doi: 10.1371/journal.pone.0131341 (PMC4497657; doi:10.1371/journal.pone.0131341)
Supplement: S1 Table — (DOCX) [file pone.0131341.s002.docx]

**S1 Table. GenBank accession numbers for all *R. microplus* OCT/Tyr receptor sequences.**

| **Sample Name** | **Accession number** | **Developmental Stage** | **Resistance status** | **Haplotype designation** |
| --- | --- | --- | --- | --- |
| 5AM3(8)A1 | KR081351 | Larvae | Resistant | - |
| 2AM3(16)A3 | KR081352 | Larvae | Resistant | - |
| 1AM3(8)A3 | KR081353 | Larvae | Resistant | - |
| 1AM5(6)A1 | KR081354 | Larvae | Resistant | - |
| 3AM2(4)A3 | KR081355 | Larvae | Resistant | - |
| H2OSample2 | KR081356 | Larvae | Susceptible | - |
| 2AM3(16)A1 | KR081357 | Larvae | Susceptible | - |
| 2AM3(16)A2 | KR081358 | Larvae | Susceptible | - |
| 1AM3(8)A2 | KR081359 | Larvae | Susceptible | - |
| 1AM3(8)A3 | KR081360 | Larvae | Susceptible | - |
| 17.1MFORG | KR051491 | Adult | Resistant | H3 |
| 65.1MFORG | KR051492 | Adult | Resistant | H4 |
| 67.2MFORG | KR051493 | Adult | Resistant | H4 |
| 26.6MFORG | KR051494 | Adult | Resistant | H3 |
| 54.1MFORG | KR051495 | Adult | Resistant | H3 |
| 51.2MFORG | KR051496 | Adult | Resistant | H3 |
| 9.1MFORG | KR051497 | Adult | Resistant | H3 |
| 49.8MFORG | KR051498 | Adult | Resistant | H3 |
| 20.2MFORG | KR051499 | Adult | Susceptible | H1 |
| 20.3MFORG | KR051500 | Adult | Susceptible | H1 |
| 44.2MFORG | KR051501 | Adult | Susceptible | H1 |
| 62.4MFORG | KR051502 | Adult | Susceptible | H1 |
| 66.2MFORG | KR051503 | Adult | Susceptible | H1 |
| 73.8MFORG | KR051504 | Adult | Susceptible | H1 |
| 86.2MFORG | KR051505 | Adult | Susceptible | H1 |
| 86.7MFORG | KR051506 | Adult | Susceptible | H1 |
| 18.2MFORG | KR051507 | Adult | Susceptible | H1 |
| 20.2-1clone | KR051508 | Adult | Susceptible | H8 |
| 20.2-3clone | KR051509 | Adult | Susceptible | H1 |
| 20.2-6clone | KR051510 | Adult | Susceptible | H1 |
| 41.12-1clone | KR051511 | Adult | Heterozygous | H6 |
| 41.12-2clone | KR051512 | Adult | Susceptible | H5 |
| 41.12-3clone | KR051513 | Adult | Heterozygous | H6 |
| 41.12-4clone | KR051514 | Adult | Heterozygous | H6 |
| 41.12-6clone | KR051515 | Adult | Susceptible | H2 |
| 70.2-1clone | KR051516 | Adult | Resistant | H3 |
| 70.2-2clone | KR051517 | Adult | Resistant | H3 |
| 70.2-3clone | KR051518 | Adult | Resistant | H3 |
| 70.2-4clone | KR051519 | Adult | Resistant | H3 |
| 70.2-5clone | KR051520 | Adult | Susceptible | H7 |
| 70.2-6clone | KR051521 | Adult | Resistant | H3 |
|  |  |  |  |  |
