# Supplementary material for: SNP Analysis Infers that Recombination Is Involved in the Evolution of Amitraz Resistance in Rhipicephalus microplus
Source: PLoS One. 2015 Jul 9;10(7):e0131341. doi: 10.1371/journal.pone.0131341 (PMC4497657; doi:10.1371/journal.pone.0131341)
Supplement: S2 Table — (DOCX) [file pone.0131341.s003.docx]

**S2 Table. Genotypes of field samples of *R. microplus* ticks at the two published SNP positions.**

| Sample | Genotype^1^ | Sample | Genotype^1^ | Sample | Genotype^1^ |
| --- | --- | --- | --- | --- | --- |
|  |  |  |  |  |  |
| 44.2MF | AA/TT | 45.1MF | AA/TT | 70.2MF | AC/TC |
| 46.2MF | AC/TC | 45.4MF | AA/TT | 7.5MF | AC/TC |
| 46.3MF | AC/TC | 45.5MF | CC/CC | 7.6MF | AA/TT |
| 46.5MF | AC/TC | 21.1MF | AA/TT | 37.2MF | AC/TC |
| 44.3MF | AA/TT | 50.3MF | AA/TT | 41.2MF | AC/TC |
| 44.5MF | AC/TC | 66.1MF | AA/TT | 41.11MF | AC/TC |
| 44.4MF | AC/TC | 66.2MF | AA/TT | 41.12MF | AC/TC |
| 71.1MF | AC/TC | 66.3MF | AC/TC | 41.13MF | AC/TC |
| 17.1MF | CC/CC | 66.5MF | CC/CC | 49.1MF | AA/TT |
| 20.1MF | AA/TT | 66.6MF | AC/TC | 49.6MF | AC/TC |
| 20.2MF | AA/TT | 67.1MF | AC/TC | 49.8MF | CC/CC |
| 20.3MF | AA/TT | 67.2MF | CC/CC | 49.10MF | AC/TC |
| 26.7MF | AC/TC | 67.3MF | AA/TT | 58.1MF | AC/TC |
| 26.8MF | AC/TC | 67.4MF | AC/TC | 58.3MF | AC/TC |
| 26.1MF | AC/TC | 67.5MF | CC/CC | 58.7MF | CC/CC |
| 26.6MF | CC/CC | 67.7MF | AA/TT | 58.11MF | CC/CC |
| 47.2MF | AC/TC | 51.1MF | AC/TC | 58.13MF | AC/TC |
| 54.1MF | CC/CC | 51.2MF | CC/CC | 93.1MF | AC/TC |
| 54.7MF | AC/TC | 51.3MF | AC/TC | 93.2MF | AC/TC |
| 54.9MF | AC/TC | 51.4MF | AC/TC | 93.3MF | AC/TC |
| 54.10MF | CC/CC | 51.5MF | CC/CC | 93.4MF | AC/TC |
| 73.1MF | CC/CC | 69.3MF | AC/TC | 93.5MF | AC/TC |
| 73.2MF | AC/TC | 69.4MF | AC/TC | 93.7MF | CC/CC |
| 73.3MF | AC/TC | 69.5MF | AC/TC | 93.8MF | AC/TC |
| 73.9MF | AC/TC | 9.2MF | CC/CC | 95.1MF | AC/TC |
| 73.4MF | AC/TC | 9.1MF | CC/CC | 95.3MF | AC/TC |
| 73.5MF | AC/TC | 9.3MF | AC/TC | 95.4MF | AC/TC |
| 73.7MF | AC/TC | 77.1MF | AC/TC | 95.6MF | AC/TC |
| 73.8MF | AA/TT | 9.6MF | CC/CC | 37.4MF | AC/TC |
| 86.1MF | AA/TT | 62.1MF | AA/TT | 40.3MM | AA/TT |
| 86.2MF | AA/TT | 62.2MF | AA/TT | 42.1MF | AC/TC |
| 86.3MF | AC/TC | 62.3MF | AA/TT | 18.2MF | AA/TT |
| 86.4MF | CC/CC | 62.4MF | AA/TT | 18.1MF | AC/TC |
| 86.5MF | AC/TC | 62.5MF | AC/TC |  |  |
| 86.6MF | AC/TC | 65.1MF | CC/CC |  |  |
| 86.7MF | AA/TT | 65.2MF | CC/CC |  |  |
| 86.8MF | AC/TC | 65.3MF | AA/TT |  |  |
| 70.1MF | AC/TC | 79.1MF | AC/TC |  |  |
|  |  |  |  |  |  |

^1^ AA/TT represents the wild type genotype, CC/CC represents the mutant resistant genotype and AC/TC represents a heterozygous form containing both wild type and mutant alleles. All samples were collected during January 2012 – April 2012.
