# Supplementary material for: SNP Analysis Infers that Recombination Is Involved in the Evolution of Amitraz Resistance in Rhipicephalus microplus
Source: PLoS One. 2015 Jul 9;10(7):e0131341. doi: 10.1371/journal.pone.0131341 (PMC4497657; doi:10.1371/journal.pone.0131341)
Supplement: S3 Table — (DOCX) [file pone.0131341.s004.docx]

**S3 Table. *Rhipicephalus microplus* larval packet test results**

| Sample number | Collection date | Tick Species | % Control at field  Concentration^2^ |
| --- | --- | --- | --- |
| 1 | 03-Mar-09 | *R. dec* | 0 |
| 2 | ?? Apr-09 | *R. dec* | 100 |
| 3 | 02-Feb-10 | *R. dec* | 100 |
| 4 | 18-Mar-10 | *R. dec* | 11.8 |
| 5 | 06-May-11 | *R. dec* | 13.2 |
| 6 | 16-Feb-11 | *R. dec* | 30 |
| 7 | 26-May-10 | *R. dec* | 100 |
| 8 | 19-Feb-13 | *R. dec* | 100 |
| 9 | 21-Feb-13 | *R. dec* | 100 |
| 10 | 30-Jan-09 | *R. dec* | 11.8 |
| 11 | 29-Apr-09 | *R. dec* | 100 |
| 12 | 25-May-10 | *R. dec* | 100 |
| 13 | 13-Apr-12 | *R. dec* | 10.9 |
| 14 | 20-Feb-09 | *R. dec* | 34.7 |
|  |  |  |  |

^2^90-100% Amitraz regarded as effective, 80-90% Effective with reservation, 50-80% Indications of developing resistance, 0-50% Indications of resistance. Amitraz concentration was at 250 ppm.
